# Poly[di-μ-thiocyanato-κ^2^ *N*:*S*;κ^2^ *S*:*N*-bis­[2-(1*H*-1,2,3-triazol-1-yl-κ*N* ^3^)pyra­zine]cadmium(II)]

**DOI:** 10.1107/S1600536809036332

**Published:** 2009-09-12

**Authors:** Cheng Qi Liu, Long Miao Xie, Ming Gen Zhao

**Affiliations:** a Department of Chemistry, Xinzhou Teachers’ University, Shanxi, Xinzhou 034000, People’s Republic of China; b Department of Chemistry, Shandong Normal University, Jinan 250014, People’s Republic of China

## Abstract

In the title two-dimensional coordination polymer, [Cd(NCS)_2_(C_6_H_5_N_5_)_2_]_*n*_, the Cd^II^ ion (site symmetry 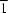) is coordinated by two N atoms from two 2-(1*H*-1,2,3-triazol-1-yl)pyrazine ligands and two N and two S atoms from four thio­cyanate anions. The N—Cd bond lengths range from 2.323 (2) to 2.3655 (19) Å and the S—Cd bond length is 2.7117 (7) Å. The associated *cisoid* angles vary from 84.99 (7) to 95.01 (7)°, indicating that the Cd^II^ ion assumes a distorted octa­hedral geometry. In the complex, each thio­cyanate anion functions as a bridging ligand, linking adjacent Cd^II^ ions with a separation of 6.4919 (6) Å, resulting in the formation of a two-dimensional sheet structure in the *bc* plane.

## Related literature

For a related crystal structure, see: Yang & Shi (2008 ▶). For the synthesis of Cd^II^ complexes with thio­cyanate anions and pyrazine derivatives as mixed bridging ligands, see: Li *et al.* (2008 ▶); Shi *et al.* (2007 ▶).
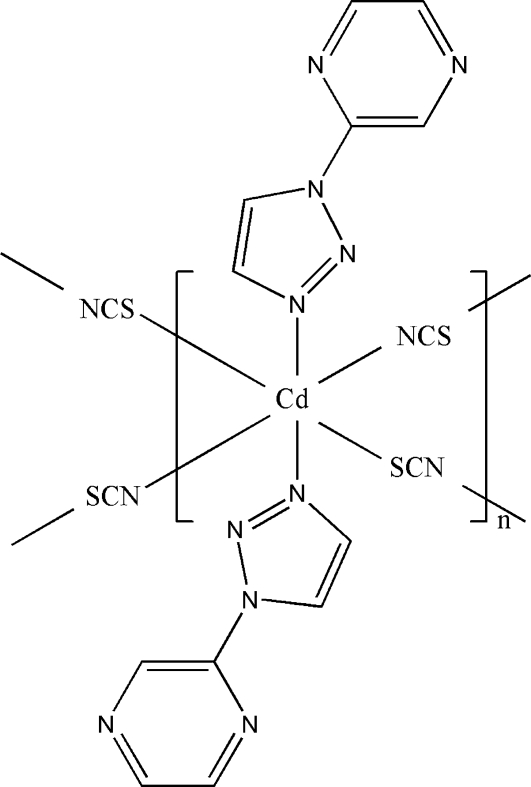


## Experimental

### 

#### Crystal data

[Cd(NCS)_2_(C_6_H_5_N_5_)_2_]
*M* *_r_* = 522.86
Monoclinic, 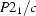

*a* = 12.5038 (15) Å
*b* = 10.7240 (13) Å
*c* = 7.3196 (9) Å
β = 106.476 (2)°
*V* = 941.2 (2) Å^3^
*Z* = 2
Mo *K*α radiation
μ = 1.41 mm^−1^
*T* = 298 K
0.24 × 0.18 × 0.16 mm

#### Data collection

Bruker SMART APEX CCD diffractometer
Absorption correction: multi-scan (*SADABS*; Sheldrick, 1996 ▶) *T*_min_ = 0.728, *T*_max_ = 0.806
5139 measured reflections
1923 independent reflections
1735 reflections with *I* > 2σ(*I*)
*R*_int_ = 0.023

#### Refinement

*R*[*F* ^2^ > 2σ(*F* ^2^)] = 0.026
*wR*(*F* ^2^) = 0.064
*S* = 1.03
1923 reflections
133 parameters
H-atom parameters constrained
Δρ_max_ = 0.55 e Å^−3^
Δρ_min_ = −0.34 e Å^−3^

### 

Data collection: *SMART* (Bruker, 1997 ▶); cell refinement: *SAINT* (Bruker, 1997 ▶); data reduction: *SAINT*; program(s) used to solve structure: *SHELXTL* (Sheldrick, 2008 ▶); program(s) used to refine structure: *SHELXTL*; molecular graphics: *SHELXTL*; software used to prepare material for publication: *SHELXTL*.

## Supplementary Material

Crystal structure: contains datablocks I, global. DOI: 10.1107/S1600536809036332/bv2122sup1.cif

Structure factors: contains datablocks I. DOI: 10.1107/S1600536809036332/bv2122Isup2.hkl

Additional supplementary materials:  crystallographic information; 3D view; checkCIF report

## Figures and Tables

**Table 1:** Selected bond angless (°)

N6^i^—Cd1—N1	84.99 (7)
N6^ii^—Cd1—N1	95.01 (7)
N6^ii^—Cd1—S1^iii^	91.07 (6)
N1—Cd1—S1^iii^	94.56 (5)
N6^ii^—Cd1—S1	88.93 (6)
N1—Cd1—S1	85.44 (5)

Symmetry codes: (i) 
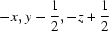
; (ii) 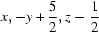; (iii) 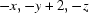.

**Fig. 1.**
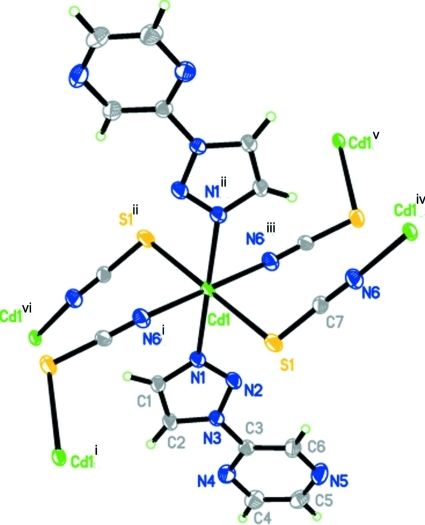
The coordination structure of (I) showing the atom numbering scheme with thermal ellipsoids drawn at the 30% probability level. [Symmetry codes: (i) -x, y - 1/2, -z + 1/2 (ii) -x, -y + 2, -z (iii) x, -y + 5/2, z - 1/2 (iv) -x, y + 1/2, -z - 1/2 (v) -x, y - 1/2, -z - 1/2 (iv) -x, y + 1/2, -z + 1/2]

**Fig. 2.**
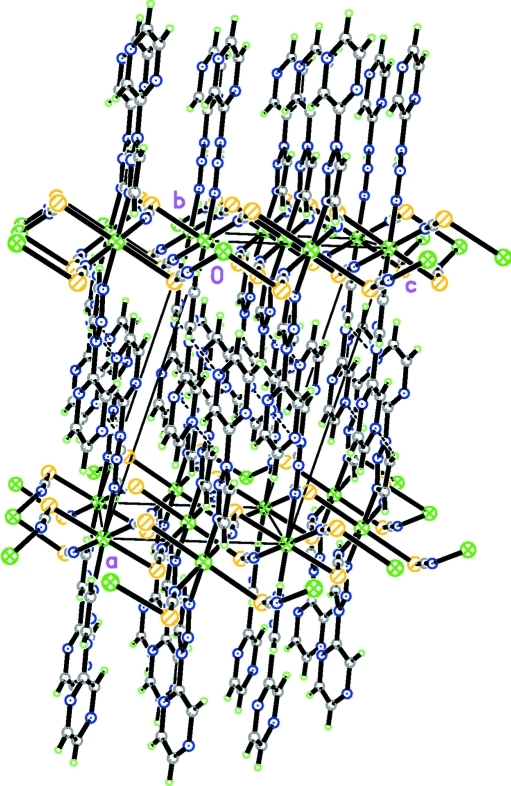
Unit cell and the part of the two-dimensional sheet on bc plane.

## supplementary crystallographic information

### Comment

2-(1*H*-1,2,3-triazol-1-yl)pyrazine is similar to 2-(pyrazol-1-yl)pyrazine (Yang & Shi 2008) in structure and therefore it should act as a bridging ligand. Our interest in synthesizing Cd^II^ complexes (Shi *et al.*, 2007; Li *et al.*, 2008) with thiocyanate anions and derivatives of pyrazine as mixed bridging ligands resulted in us selecting thiocyanato and 2-(1*H*-1,2,3-triazol-1-yl)pyrazine as ligands, but only the title complex was obtained, in which 2-(1*H*-1,2,3-triazol-1-yl)pyrazine only functions as a terminal ligand. Herein we report the crystal structure of the title complex.

The asymmetric unit and symmetry-related fragments of (I) are shown in Fig. 1, and Fig.1 and Table 1 reveal that Cd1 atom is in a distorted octahedral CdN_4_S_2_ coordination geometry. In the crystal each Cd^II^ ion is surrounded by four other symmetry-related Cd^II^ ions with separation with 6.4919 (6) Å and the adjacent Cd^II^ ions were bridged by one thiocyanato anions and it forms a two-dimensional sheet on *bc* plane as shown in Fig. 2. 2-(1*H*-1,2,3-triazol-1-yl)pyrazine only acts as a monodentate ligand.

### Experimental

An 8 ml methanol solution of 2-(1*H*-1,2,3-triazol-1-yl)pyrazine (0.0401 g, 0.272 mmol), 5 ml water solution of Cd(ClO_4_)_2_.6H_2_O (0.1120 g, 0.267 mmol) and 5 ml water solution of NaSCN (0.0435 g, 0.537 mmol) were mixed together and stirred for a few minutes. The colorless single crystals were obtained after the filtrate had been allowed to stand at room temperature for ten days.

### Refinement

All H atoms were placed in calculated positions and refined as riding with C—H = 0.93 Å and *U*_iso_(H) = 1.2*U*_eq_(C).

### Crystal data

**Table:**

[Cd(NCS)_2_(C_6_H_5_N_5_)_2_]	*F*(000) = 516
*M**_r_* = 522.86	*D*_x_ = 1.845 Mg m^−^^3^
Monoclinic, *P*2_1_/*c*	Mo *K*α radiation, λ = 0.71073 Å
Hall symbol: -P 2ybc	Cell parameters from 3160 reflections
*a* = 12.5038 (15) Å	θ = 2.6–28.0°
*b* = 10.7240 (13) Å	µ = 1.41 mm^−^^1^
*c* = 7.3196 (9) Å	*T* = 298 K
β = 106.476 (2)°	Block, colorless
*V* = 941.2 (2) Å^3^	0.24 × 0.18 × 0.16 mm
*Z* = 2	

### Data collection

**Table:**

Bruker SMART APEX CCD diffractometer	1923 independent reflections
Radiation source: fine-focus sealed tube	1735 reflections with *I* > 2σ(*I*)
graphite	*R*_int_ = 0.023
φ and ω scans	θ_max_ = 26.4°, θ_min_ = 1.7°
Absorption correction: multi-scan (*SADABS*; Sheldrick, 1996)	*h* = −15→15
*T*_min_ = 0.728, *T*_max_ = 0.806	*k* = −13→12
5139 measured reflections	*l* = −6→9

### Refinement

**Table:**

Refinement on *F*^2^	Primary atom site location: structure-invariant direct methods
Least-squares matrix: full	Secondary atom site location: difference Fourier map
*R*[*F*^2^ > 2σ(*F*^2^)] = 0.026	Hydrogen site location: inferred from neighbouring sites
*wR*(*F*^2^) = 0.064	H-atom parameters constrained
*S* = 1.03	*w* = 1/[σ^2^(*F*_o_^2^) + (0.0289*P*)^2^ + 0.6077*P*] where *P* = (*F*_o_^2^ + 2*F*_c_^2^)/3
1923 reflections	(Δ/σ)_max_ = 0.001
133 parameters	Δρ_max_ = 0.55 e Å^−^^3^
0 restraints	Δρ_min_ = −0.34 e Å^−^^3^

### Special details

**Table:**

Geometry. All e.s.d.'s (except the e.s.d. in the dihedral angle between two l.s. planes) are estimated using the full covariance matrix. The cell e.s.d.'s are taken into account individually in the estimation of e.s.d.'s in distances, angles and torsion angles; correlations between e.s.d.'s in cell parameters are only used when they are defined by crystal symmetry. An approximate (isotropic) treatment of cell e.s.d.'s is used for estimating e.s.d.'s involving l.s. planes.
Refinement. Refinement of *F*^2^ against ALL reflections. The weighted *R*-factor *wR* and goodness of fit *S* are based on *F*^2^, conventional *R*-factors *R* are based on *F*, with *F* set to zero for negative *F*^2^. The threshold expression of *F*^2^ > σ(*F*^2^) is used only for calculating *R*-factors(gt) *etc*. and is not relevant to the choice of reflections for refinement. *R*-factors based on *F*^2^ are statistically about twice as large as those based on *F*, and *R*- factors based on ALL data will be even larger.

### Fractional atomic coordinates and isotropic or equivalent isotropic displacement parameters (Å^2^)

**Table:**

	*x*	*y*	*z*	*U*_iso_*/*U*_eq_	
C1	0.1593 (2)	0.7435 (2)	0.0075 (4)	0.0390 (6)	
H1	0.0980	0.6904	−0.0225	0.047*	
C2	0.2671 (2)	0.7082 (2)	0.0439 (4)	0.0405 (6)	
H2	0.2951	0.6278	0.0441	0.049*	
C3	0.44202 (19)	0.8353 (2)	0.1282 (3)	0.0348 (5)	
C4	0.6101 (2)	0.7616 (3)	0.1322 (4)	0.0521 (7)	
H4	0.6555	0.6987	0.1083	0.063*	
C5	0.6582 (2)	0.8710 (3)	0.2116 (4)	0.0530 (7)	
H5	0.7347	0.8817	0.2350	0.064*	
C6	0.4896 (2)	0.9434 (3)	0.2156 (5)	0.0506 (7)	
H6	0.4446	1.0041	0.2468	0.061*	
C7	0.08801 (19)	1.2249 (2)	0.3671 (3)	0.0331 (5)	
Cd1	0.0000	1.0000	0.0000	0.03129 (10)	
N1	0.15559 (16)	0.86932 (18)	0.0222 (3)	0.0370 (5)	
N2	0.25664 (17)	0.91457 (18)	0.0669 (3)	0.0393 (5)	
N3	0.32500 (16)	0.81662 (17)	0.0798 (3)	0.0336 (4)	
N4	0.50015 (18)	0.7423 (2)	0.0879 (4)	0.0464 (5)	
N5	0.5982 (2)	0.9623 (3)	0.2560 (5)	0.0615 (7)	
N6	0.07295 (19)	1.32994 (19)	0.3793 (3)	0.0452 (5)	
S1	0.11190 (7)	1.07527 (6)	0.35667 (11)	0.0530 (2)	

### Atomic displacement parameters (Å^2^)

**Table:**

	*U*^11^	*U*^22^	*U*^33^	*U*^12^	*U*^13^	*U*^23^
C1	0.0328 (13)	0.0276 (11)	0.0548 (16)	0.0003 (9)	0.0097 (11)	−0.0064 (11)
C2	0.0363 (13)	0.0268 (11)	0.0566 (16)	0.0025 (10)	0.0104 (12)	−0.0060 (11)
C3	0.0333 (12)	0.0363 (12)	0.0350 (12)	0.0027 (10)	0.0098 (10)	−0.0011 (10)
C4	0.0379 (15)	0.0618 (18)	0.0593 (19)	0.0058 (13)	0.0181 (13)	−0.0065 (15)
C5	0.0335 (14)	0.0681 (19)	0.0564 (18)	−0.0022 (13)	0.0113 (13)	0.0008 (15)
C6	0.0370 (14)	0.0413 (15)	0.070 (2)	0.0015 (12)	0.0094 (13)	−0.0099 (14)
C7	0.0310 (12)	0.0315 (12)	0.0370 (13)	−0.0020 (9)	0.0097 (10)	−0.0056 (10)
Cd1	0.02776 (14)	0.01935 (13)	0.04655 (17)	0.00164 (8)	0.01019 (11)	0.00206 (9)
N1	0.0321 (10)	0.0283 (10)	0.0496 (13)	0.0032 (8)	0.0098 (9)	−0.0009 (9)
N2	0.0340 (11)	0.0265 (10)	0.0578 (14)	0.0039 (8)	0.0134 (10)	−0.0011 (9)
N3	0.0311 (10)	0.0274 (9)	0.0415 (11)	0.0041 (8)	0.0087 (8)	−0.0024 (8)
N4	0.0379 (12)	0.0450 (12)	0.0575 (15)	0.0042 (10)	0.0157 (10)	−0.0089 (11)
N5	0.0397 (14)	0.0541 (14)	0.085 (2)	−0.0095 (12)	0.0082 (13)	−0.0145 (15)
N6	0.0462 (13)	0.0323 (11)	0.0571 (14)	0.0036 (9)	0.0145 (11)	−0.0081 (10)
S1	0.0712 (5)	0.0266 (3)	0.0485 (4)	0.0086 (3)	−0.0037 (3)	−0.0046 (3)

### Geometric parameters (Å, °)

**Table:**

C1—C2	1.352 (3)	C6—N5	1.322 (4)
C1—N1	1.355 (3)	C6—H6	0.9300
C1—H1	0.9300	C7—N6	1.150 (3)
C2—N3	1.356 (3)	C7—S1	1.638 (2)
C2—H2	0.9300	Cd1—N6^i^	2.323 (2)
C3—N4	1.316 (3)	Cd1—N6^ii^	2.323 (2)
C3—C6	1.375 (4)	Cd1—N1	2.3655 (19)
C3—N3	1.419 (3)	Cd1—N1^iii^	2.3655 (19)
C4—N4	1.336 (4)	Cd1—S1^iii^	2.7117 (7)
C4—C5	1.370 (4)	Cd1—S1	2.7117 (7)
C4—H4	0.9300	N1—N2	1.306 (3)
C5—N5	1.328 (4)	N2—N3	1.341 (3)
C5—H5	0.9300	N6—Cd1^iv^	2.323 (2)
			
C2—C1—N1	108.6 (2)	N6^ii^—Cd1—N1^iii^	84.99 (7)
C2—C1—H1	125.7	N1—Cd1—N1^iii^	180.0
N1—C1—H1	125.7	N6^i^—Cd1—S1^iii^	88.93 (6)
C1—C2—N3	104.2 (2)	N6^ii^—Cd1—S1^iii^	91.07 (6)
C1—C2—H2	127.9	N1—Cd1—S1^iii^	94.56 (5)
N3—C2—H2	127.9	N1^iii^—Cd1—S1^iii^	85.44 (5)
N4—C3—C6	123.3 (2)	N6^i^—Cd1—S1	91.07 (6)
N4—C3—N3	115.7 (2)	N6^ii^—Cd1—S1	88.93 (6)
C6—C3—N3	121.0 (2)	N1—Cd1—S1	85.44 (5)
N4—C4—C5	122.2 (3)	N1^iii^—Cd1—S1	94.56 (5)
N4—C4—H4	118.9	S1^iii^—Cd1—S1	180.0
C5—C4—H4	118.9	N2—N1—C1	109.69 (19)
N5—C5—C4	121.6 (3)	N2—N1—Cd1	121.04 (14)
N5—C5—H5	119.2	C1—N1—Cd1	129.10 (16)
C4—C5—H5	119.2	N1—N2—N3	106.19 (18)
N5—C6—C3	121.0 (3)	N2—N3—C2	111.33 (19)
N5—C6—H6	119.5	N2—N3—C3	119.93 (19)
C3—C6—H6	119.5	C2—N3—C3	128.7 (2)
N6—C7—S1	178.2 (3)	C3—N4—C4	115.1 (2)
N6^i^—Cd1—N6^ii^	180.0	C6—N5—C5	116.6 (3)
N6^i^—Cd1—N1	84.99 (7)	C7—N6—Cd1^iv^	152.8 (2)
N6^ii^—Cd1—N1	95.01 (7)	C7—S1—Cd1	106.59 (9)
N6^i^—Cd1—N1^iii^	95.01 (7)		

Symmetry codes: (i) −*x*, *y*−1/2, −*z*+1/2; (ii) *x*, −*y*+5/2, *z*−1/2; (iii) −*x*, −*y*+2, −*z*; (iv) −*x*, *y*+1/2, −*z*+1/2.
